# Comparative Evaluation of Different Obturation Techniques for Root Canal Filling of Permanent Teeth: An In-Vitro Study

**DOI:** 10.7759/cureus.68623

**Published:** 2024-09-04

**Authors:** Adhishree S Chib, Neeta S Padmawar, Sonali Waghmare, Durgesh A Tiwari, Shahinwaz Mulani, Megna Bhatt

**Affiliations:** 1 Department of Pediatric Dentistry, Consultant Pedodontics, Muskaan Dentals Global, Gurugram, IND; 2 Department of Pediatric Dentistry, Rural Dental College, Pravara Institute of Medical Sciences, Loni, IND; 3 Department of Oral Pathology, YMT Dental College and Hospital, Kharghar, Navi Mumbai, IND; 4 Department of Conservative Dentistry and Endodontics, Yogita Dental College and Hospital, Khed, IND; 5 Department of Prosthodontics, Guru Govind Singh Dental College and Research Center, Burhanpur, IND; 6 Department of Conservative Dentistry and Endodontics, Shree Bankey Bihari Dental College and Research Centre, Ghaziabad, IND

**Keywords:** gutta-percha, premolars, root canal obturation, root canal filling materials, stereomicroscope

## Abstract

Introduction: Achieving success in root canal (RC) therapy relies on three key components: comprehensive cleaning of the canal, efficient disinfection, and proper filling of the canal space. The present study aimed to compare the efficacy of four obturation techniques: single cone (SC), GuttaCore (GC), cold lateral condensation (LC), and C Point system for RC filling of permanent teeth.

Materials and methods: 76 extracted human mandibular first premolars were divided into four groups, with 19 teeth in each group (group A, obturation with SC technique; group B, obturation with GC; group C, obturation with LC; and group D, obturation with C Point system). The samples were marked at 4 mm and 8 mm from the root apex using a marker and calliper, sectioned horizontally, and analyzed under a stereomicroscope at 20x magnification. The mean percentage (%) of gutta-percha (GP)-filled area was compared using a two-way analysis of variance (ANOVA), followed by the Bonferroni post-hoc test.

Results: The mean percentage of GP-filled area at a distance of 4 mm from the apex was highest in group B (0.86±0.04; 95% CI: 0.845-0.881), followed by group D (0.70±0.07; 95% CI: 0.664-0.736), group A (0.61±0.05; 95% CI: 0.595-0.642), and least in group C (0.58±0.09; 95% CI: 0.543-0.627), and the difference was statistically significant (p≤0.05). The post-hoc pairwise comparison of groups at 4 mm revealed that there were statistically significant differences between group D and groups B, C, and A (p ≤ 0.001). The mean percentage of the GP area at a distance of 8 mm from the apex was highest in group B (0.81±0.10; 95% CI: 0.761-0.861), followed by group D (0.75±0.07; 95% CI: 0.725-0.792), group A (0.69±0.07; 95% CI: 0.658-0.729), and lowest in group C (0.65±0.10; 95% CI: 0.607-0.709), and this difference was statistically significant (p≤0.05). Post-hoc pairwise comparison of groups at 8 mm revealed that there was a statistically significant difference between groups D and C (p=0.006), whereas no statistically significant differences were noted between groups D and B (p=0.473). Furthermore, no statistically significant differences were noted between groups C and A at 4 mm and 8 mm (p>0.05).

Conclusion: Obturation with the GC system provided the best results in terms of the percentage of GP-filled area at 4 mm. However, at 8 mm from the apical region, both the GC and C Point systems provided similar results.

## Introduction

Achieving thorough filling of the root canal (RC) system is considered a crucial element for the favorable outcome of endodontic therapy [1]. Throughout the evolution of endodontic practice, various strategies have been devised to accomplish the objective of establishing a hermetic seal within RC. Among these methods are the classic lateral compaction technique, the single-cone (SC) technique, and approaches employing thermoplasticized gutta-percha (GP) materials [2].

The traditional method of obturation, known as cold lateral condensation (LC), is commonly used in endodontics. This particular approach entails the placement of a single GP cone combined with a sealer within the prepared RC, followed by the incorporation of additional GP cones that are consolidated through the utilization of a spreader [3]. The notion of heating GP to achieve consistent three-dimensional obturation was first introduced by Schilder during the 1960s. The objective was to develop a method that would yield a uniform, durable, and compatible substance that conforms to the intricate and diverse anatomy of the RC system. This approach involves compacting the heated GP within the canal to mold it onto the shaped walls of the RC. This technique minimizes the use of sealer [4]. According to a systematic review by Bhandi et al., it was concluded that thermoplasticized GP methods demonstrated superior results, notwithstanding a potential bias towards LC due to the learning curve [5].

A novel core-carrier system, known as the GuttaCore (GC) technique, was introduced, utilizing cross-linked thermoset GP to replace Vectra or polysulfone plastic carriers in Thermafil Plus. This innovation, developed by Dentsply’s GC, facilitates removal of the carrier (obturator) during retreatment [6]. In a study by Li et al., it was concluded that the GC group exhibited notably reduced occurrences of gaps and voids compared with the LC group [7].

The C Point system, which follows the principle of hydraulic condensation, consists of a novel hygroexpandable nylon polymer-based SC RC filling material (C Point), which has been recently introduced. The composition included a central polyamide core consisting of two nylon polymers, with the outer layer being a cross-linked copolymer of acrylonitrile and vinylpyrrolidone. Upon exposure to moisture, the external layer of C Point demonstrates radial non-isotropic expansion, facilitating the movement of the accompanying sealer towards the canal wall to effectively conform to irregularities within the RC [8]. Yadav et al. conducted a study comparing the obturation quality of three techniques: LC, GC, and C Point systems, and it was concluded that among the three methods that were analyzed, GC demonstrated the most favorable outcomes with regard to the quality of RC filling. On the other hand, the C Point system is linked to internal flaws such as tears and delamination, which could potentially have a negative impact on the system's long-term effectiveness [9].

Due to the lack of sufficient literature on the C Point and GC systems, the present study was conducted to compare the percentage (%) of GP-filled area in mandibular first premolars obturated with the C Point, GC, LC, and SC techniques. The null hypothesis posited for the study was that there would not be any significant differences between the groups in the mean percentage of GP-filled areas.

## Materials and methods

Study design

This in vitro experimental study was carried out in the Department of Conservative Dentistry and Endodontics with prior approval from the Institutional Ethical Committee (EC/NEW/INST/2022/2512/2024-034). The study adhered to the principles outlined in the Declaration of Helsinki, and written informed consent was obtained from the patients to use their extracted tooth samples.

Sample size calculation

The sample size was calculated using the G*Power software version 3.1. Power analysis indicated that a total sample size of 76 was required to achieve a study power of 80% with an alpha error of 5% and an effect size of 0.4 [10]. The study included four groups, each comprising 19 extracted teeth.

Methodology

A total of 76 extracted human mandibular first premolars with fully formed root apices, extracted for orthodontic reasons, were collected for the study. All teeth were single-rooted with a straight, single canal, as verified by radiographic examination. The selection criteria for inclusion were teeth with a root length of 10-15 mm, free from external and internal resorption, cracks, craze lines, calcification, caries, fractures, previous restorations, prior RC treatment, or the presence of multiple roots or canals.

The teeth were decontaminated and immersed in 0.05 a sodium chloramine solution (Otto-Chemie, Mumbai) at ambient temperature. The crowns of the teeth were excised 1 mm above the cementoenamel junction (CEJ) using a diamond disc at a slow pace (Strauss Liberal, India) while being sprayed with water. The patency of the RC was verified using a #10 K-file manufactured by Mani in India, whereas the working length was ascertained by introducing a #15 K-file from the same manufacturer into the RC until its tip was barely discernible at the apical foramen, after which the length was decreased by 1 mm to determine the precise working length.

RCs of the specimens were prepared using a ProTaper rotary file system (Dentsply, India) up to F4. The canals were then irrigated with 2 ml of 17% ethylenediaminetetraacetic acid (EDTA) (Fisher Scientific, India), 2 ml of 5.25% sodium hypochlorite (Fisher Scientific, India), and 4 ml of saline. The canals were subsequently dried with paper points (Dentsply, India) and randomly allocated into four groups, each consisting of 19 samples (n = 19) (Group A, Group B, Group C, and Group D). In group A (SC technique), an F4 SC GP (Dentsply, India) was coated with AH-26 sealer and inserted into the canal. The surplus GP present at the coronal opening was removed, and the coronal portion of the tooth was carefully compacted using an endodontic plugger; Group B (GC), where the GC obturator (Dentsply Sirona) (size 40/.04) was heated in a ThermaPrep oven, and sealer was applied using a Lentulospiral. The obturator was immediately inserted into its working length. Excess GP was removed with a heated plugger, and the placement handle was detached at the orifice by twisting; group C (LC), where a #40 GP (Dentsply, India) cone with a 0.02 taper was inserted into the canal up to the working length, and the master cone was verified radiographically. Following placement of the master cone in the RC, accessory GP points (#25/0.02) were inserted into the spaces created by the spreader immediately after its removal. The excess GP was trimmed at the CEJ, and the coronal portion was gently condensed using an endodontic plugger. In Group D (C Point system), an F4 C Point (EndoTechnologies, USA) with a confirmed tug-back was selected. After being thinly covered with a sealer, the instrument was placed into the RC, which had been previously prepared, until it reached the desired depth. Any surplus material was eliminated from the canal opening using a bur attached to a slow-speed handpiece and was operated without the use of water.

Obturation was confirmed radiographically. The access cavities of the specimens were sealed with type II glass ionomer cement. The teeth were then incubated at 37°C and 100% humidity for 14 days to ensure complete setting of the sealers. The samples were marked at 4 and 8 mm from the root apex using a marker and calliper, sectioned horizontally, and analyzed under a stereomicroscope at 20x magnification. Images were captured using a Coolpix S2500 camera (Nikon, Tokyo, Japan). Area measurements were conducted using ImageJ analysis software to record the area of the canal and filling material. The percentage of GP-filled area along the canal wall was calculated. A stereomicroscope was used in the present study for assessment, as while newer techniques such as micro-CT or cone-beam computed tomography (CBCT) provide excellent imaging, they can be expensive and not always available in all dental practices. A stereomicroscope is a more accessible and cost-effective tool for routine assessment. Many dental professionals are trained and familiar with using stereomicroscopes. The skill and experience they have with this tool make it a reliable choice for assessing the quality of root canal fillings. In summary, the stereomicroscope remains a valuable tool due to its ability to provide high-resolution, real-time, three-dimensional visualization, making it a practical choice for many practitioners in root canal GP assessment. The evaluations were done at 4 mm and 8 mm, as these specific measurements allowed for a targeted assessment of the most critical areas in the root canal system. The focus was on areas that are most prone to complications, such as inadequate cleaning or shaping, which can lead to treatment failure.

Statistical analysis

The data were organized in Microsoft Excel and analyzed using IBM Corp. Released 2015. IBM SPSS Statistics for Windows, Version 23.0. Armonk, NY: IBM Corp. The normality of the data was assessed using the Shapiro-Wilk test, and the data were found to be normally distributed. Parametric data is presented as mean ± standard deviation (SD). The mean % of the GP-filled area was compared using a two-way analysis of variance (ANOVA) followed by the Bonferroni post-hoc test. The level of significance was set at p ≤ 0.05.

## Results

**Figure 1:**
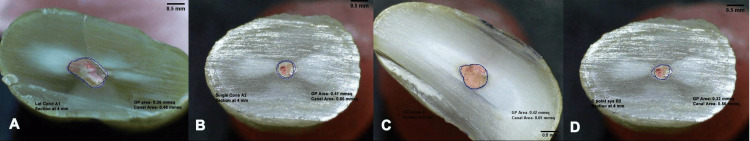
Stereomicroscopic analysis (20x magnification) of gutta-percha area at 4 mm in obturation technique [A] Cold lateral condensation, [B] Single cone, [C] GuttaCore, [D] C Point system

The null hypothesis was rejected in our study, as statistically significant differences were observed between the groups. The mean % of GP area at a distance of 4 mm from the apex was highest in group B (0.86±0.04; 95% CI: 0.845-0.881), followed by group D (0.70±0.07; 95% CI: 0.664-0.736), group A (0.61±0.05; 95% CI: 0.595-0.642), and least in group C (0.58±0.09; 95% CI: 0.543-0.627). There were statistically significant differences between the groups (p=0.001). An effect size of 0.74 indicated a moderate to large effect, which suggested that this finding had a meaningful and substantial impact, which could be of practical importance (Table 1) (Figure 1).

**Table 1:** Comparison of percentage (%) of gutta percha (GP) area in groups (n=76) by two-way ANOVA test at 4 mm *p<0.05: Significant; SD: Standard deviation; Data presented in form of Mean±SD

Groups	% of GP area (Mean±SD)	Confidence interval at 95%		p-value	Effect size
		Upper bound	Lower bound		
Group A	0.61±0.05	0.642	0.595	0.001*	0.74
Group B	0.86±0.04	0.881	0.845		
Group C	0.58±0.09	0.627	0.543		
Group D	0.70±0.07	0.736	0.664		

The post-hoc pairwise comparison of groups at 4 mm revealed that there were statistically significant differences between group D and groups B, C, and A (p ≤ 0.001). Group B also showed statistically significant differences between groups C and A (p < 0.001), whereas no statistically significant difference was observed between groups C and A (p=0.692) (Table 2).

**Table 2:** Post-hoc pairwise comparisons by Bonferroni test at 4 mm *p<0.05: Significant; SE: Standard error

Groups	Comparison groups	Mean difference	SE	t value	p-value
Group A	Group B	0.245	0.021	11.583	0 .001*
	Group C	-0.034	0.021	-1.594	0.692
	Group D	0.082	0.021	3.861	0.001*
Group B	Group C	0.278	0.021	13.178	0 .001*
	Group D	-0.163	0.021	-7.722	0 .001*
Group C	Group D	0.115	0.021	5.455	0 .001*

**Figure 2:**
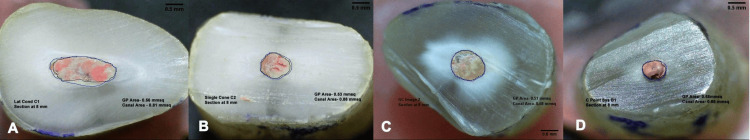
Stereomicroscopic analysis (20x magnification) of gutta-percha area at 8 mm in obturation technique [A] Cold lateral condensation, [B] Single cone, [C] GuttaCore, [D] C point system

The mean percentage of GP area at a distance of 8 mm from the apex was highest in group B (0.81±0.10; 95% CI: 0.761-0.861), followed by group D (0.75±0.07; 95% CI: 0.725-0.792), group A (0.69±0.07; 95% CI: 0.658-0.729), and least in group C (0.65±0.10; 95% CI: 0.607-0.709). There were statistically significant differences between the groups (p=0.001). An effect size of 0.31 suggested statistical significance; however, it may not always be practically significant. This indicated a small-to-moderate effect (Table 3) (Figure 2).

**Table 3:** Comparison of percentage (%) of gutta percha (GP) area in groups (n=76) by two-way ANOVA test at 8 mm *p<0.05: Significant; SD: Standard deviation; Data presented in form of Mean±SD

Groups	% of GP area (Mean±SD)	Confidence interval at 95%		p-value	Effect size
		Upper bound	Lower bound		
Group A	0.69±0.07	0.729	0.658	0.001*	0.31
Group B	0.81±0.10	0.861	0.761		
Group C	0.65±0.10	0.709	0.607		
Group D	0.75±0.07	0.792	0.725		

The post-hoc pairwise comparison of groups at 8 mm revealed that there was a statistically significant difference between groups D and C (p=0.006), whereas no statistically significant differences were noted between groups D and B (p=0.473) or group A (p=0.172). Group B also showed statistically significant differences between groups C and A (p < 0.001), whereas no statistically significant difference was observed between groups C and A (p=1) (Table 4).

**Table 4:** Post-hoc pairwise comparisons by Bonferroni test at 8 mm *p<0.05: Significant; SE: Standard error

Groups	Comparison groups	Mean difference	SE	t value	p-value
Group A	Group B	0.117	0.029	4.016	0 .001*
	Group C	-0.036	0.029	-1.225	1
	Group D	0.065	0.029	2.233	0.172
Group B	Group C	0.153	0.029	5.241	0 .001*
	Group D	-0.052	0.029	-1.783	0.473
Group C	Group D	0.101	0.029	3.458	0.006*

## Discussion

In the present study, the percentage of GP-filling area was used to estimate the quality of RC filling using four techniques: SC (group A), GC (group B), LC (group C), and C Point System (group D). Earlier apical or coronal leakage was used to determine the quality of RC; however, the clinical significance of these methods was doubtful [11]. Therefore, a high-quality RC filling should consist of the highest possible percentage of GP-filled area.

The results of the present study indicated that the GC method provided the highest mean percentage of GP-filled area, followed by the C Point system at the 4 mm and 8 mm levels. The results of the LC and SC techniques were comparable and provided less percentage of the GP-filled area compared with the GC and C Point methods of obturation. These results are consistent with those of previous studies [7,9,10]. Despite undergoing hygroscopic expansion, Point C did not successfully create a tight seal at depths of 4 and 8 mm, showing a significant reliance on the sealer used.

GC utilizes a core made of cross-linked GP, which serves as a central carrier surrounded by thermoplasticized GP material. Through this design, the material can flow more evenly within the RC system in three dimensions, encompassing both accessory and lateral canals. The thermoplasticized GP component within the GC exhibited a soft and pliable nature upon heating, enabling it to conform more precisely to the irregularities present on the walls of the RC. Consequently, this adaptive quality leads to the creation of a superior seal and a more thorough filling of the canal. In contrast, techniques such as SC and LC do not incorporate extensive thermoplasticization processes, thereby limiting their adaptability to intricate canal walls. In the SC technique, the GP is commonly inserted into the canal without a carrier, potentially resulting in a less consistent filling. On the other hand, LC entails the compression of GP cones against the canal walls, yet this approach may give rise to gaps if the pressure is not uniformly distributed during the procedure [9,10,12].

In the present study, no statistically significant differences were noted in the mean percentage of the GP-filled area between the SC technique and LC. Similar results were reported in a systematic review by Barcelos et al. [13]. This phenomenon may have occurred because of the challenge posed by the non-uniform distribution of sealer when using the SC technique, stemming from the inherent difficulty in precisely controlling the sealer volume encasing the GP cone. This leads to suboptimal adaptation of the sealer, which is crucial for procedure success. An alternative explanation is the intricate placement of a singular round cone within a canal that may have irregular contours, thereby hindering a gap-free fit. Moreover, enhancing the quality of the filling accomplished through the SC technique is feasible by using endodontic sealers characterized by superior dimensional stability, such as those based on epoxy resin, or those exhibiting hygroscopic expansion, such as calcium silicate-based sealers, instead of zinc oxide and eugenol-based sealers known to contract post-setting, thus compromising sealing integrity [14,15]. During the LC process, it was observed that GP cones tend to be tightly compacted together in the RC space, although they maintain their individual integrity. This phenomenon has been documented in various studies [16,17], indicating the importance of proper cone placement for successful treatment outcomes. Moreover, the cold GP material does not exhibit strong adhesion properties to the RC walls, which can lead to displacement of the material during the compaction phase of the treatment.

The effect size in the present study revealed that the results were more practically significant at 4 mm than 8 mm from the apex. This could have been due to the fact that the apical 4 mm segment of the RC system is commonly characterized by a greater degree of complexity, displaying a heightened prevalence of accessory canals, lateral canals, and apical delta structures. The intricate nature of the root canal system in this specific region poses increased challenges when it comes to thorough cleaning, shaping, and filling of the canal, ultimately leading to more significant repercussions when alterations are made to obturation techniques. Conversely, when we consider a distance of 8 mm from the apex, the canal anatomy tends to exhibit a lower level of intricacy, with a larger and more uniform space. As a result of this simplified anatomy, the influence of various techniques employed in this area may not be as pronounced, consequently yielding a diminished effect size in comparison to the apical 4 mm segment [18].

The present study evaluated the results after two weeks of storage. According to Liu et al. [19], after 54 months of obturation, fewer voids were noticed with warm condensation compared to the SC technique. According to Zhang et al. [20], it was concluded that the efficiency of retreatment in the oval-shaped canal was closely related to the storage time rather than the filling technique using a tricalcium silicate sealer.

Limitations

The present study evaluated the percentage of GP-filled areas using stereomicroscopy; however, more advanced techniques such as micro-computed tomography could be used for better evaluation. Moreover, the presence of voids and the percentage of sealers were not evaluated in this study. The small sample size is another limitation of the present study. The present study took two weeks as storage time, which helped to provide immediate or short-term outcomes of root canal treatments. However, extended storage durations for evaluating root-filled teeth offer a more comprehensive and clinically relevant assessment of sealer setting and root-filling quality. Therefore, future research should evaluate the efficacy of different obturation techniques in RC filling using advanced technology, a larger sample size, and an extended storage time of at least six months.

## Conclusions

Within the confines of this particular investigation, it can be deduced that the RC obturated with the GC technique displayed the highest percentage of GP-filled canals along with the C Point system evaluated at a distance of 8 mm from the apex. Nonetheless, when evaluated at 4 mm, the distinctive feature of hygroscopic radial expansion in the C Point system did not result in an enhanced quality of RC filling. At both 4 mm and 8 mm intervals, there were no statistically notable variances between employing the SC method and the LC technique, indicating a lower percentage of GP-filled canals in comparison to the GC and C Point systems. Based on the findings of the present study, the GC obturation system can be used as an effective obturation technique for RC.
